# Preparation of Emulsifier-Free Styrene–Acrylic Emulsion via Reverse Iodine Transfer Polymerization

**DOI:** 10.3390/polym13193348

**Published:** 2021-09-29

**Authors:** Tao Huang, Shuling Gong

**Affiliations:** College of Chemistry and Molecular Sciences, Wuhan University, Wuhan 430072, China; 2017102030109@whu.edu.cn

**Keywords:** styrene–acrylic emulsion, cooperated, reverse iodine transfer polymerization, polymeric emulsifier

## Abstract

Styrene–acrylic emulsions containing hydroxyl functional monomer unit’s component are widely used for maintenance coating. In this paper, a stable emulsifier-free styrene–acrylic emulsion with solid content over 43% could be obtained in 210 min via reverse iodine transfer polymerization (RITP). By adding a mixture of methacrylic acid (MAA) and poly(ethylene glycol)methyl ether methacrylate (PEGMA) into a system containing a high content of hydroxyl functional monomer component (19.4 wt.% of the total monomer mass), styrene (St) could be copolymerized with methyl methacrylate (MMA); the modified film exhibited good hardness properties, good adhesive properties, and low water absorption. An increase in the amount of PEGMA decreased the glass transition temperature (*T*_g_). When 1.4 times the reference amount of initiator was added, the highest molecular weight *M*_n_ could reach 40,000 g.·mol^−1^ with 0.25 times the reference amount of iodine in the emulsion. The largest tensile strength of the dried emulsion film over 5.5 MPa endowed the material with good mechanical properties. Living polymerization was proven by the kinetics of RITP emulsion and chain extension reaction. TEM micrographs manifest the emulsification of the seed random copolymer. This paper may provide a potential methodology for preparing polymer materials with excellent mechanical properties.

## 1. Introduction

Styrene–acrylic emulsions are widely used as industrial maintenance coating for the acrylate unit’s resistance to photodegradation and the styrene unit’s resistance to hydrolysis [1,2]. Furthermore, styrene–acrylic emulsions are also used for preparing nanoparticles [3,4,5,6,7,8,9], which are applied in the treatment of bacterial infections [10,11] or encapsulation medicine [12,13]. The optionality of the monomer endows emulsion polymers with some special properties. In emulsion polymerization, methacrylic Acid (MAA), methacrylic acid-β-hydroxyethyl ester (HEMA), methyl methacrylate (MMA), styrene (St), *n*-butyl acrylate (BA), and *n*-butyl methacrylate (BMA) are widely used monomers [14,15]. For example, the carboxyl group from MAA units or acrylic acid (AA) units endows the polymer with adhesive properties [16]; the hydroxyl group from methacrylic acid (MAA) units or methacrylic acid-β-hydroxyethyl ester (HEMA) can be crosslinked with amino resin [17], whereby the modified film with the crosslinked structure exhibits good mechanical properties [18]. MMA or St are used as hard monomers, which can increase the glass transition temperature of the polymer [1]. BA is used as a soft monomer [19]. Thus, styrene–acrylic emulsion polymers with functional groups such as carboxyl or hydroxyl in the pendant group have wide application prospects.

Styrene–acrylic emulsions can be prepared via emulsion polymerization with or without an emulsifier. Emulsion polymerization with a nonpolymeric emulsifier is conducted via the polymerization of weak water-soluble monomers within the emulsion of a nonpolymeric emulsifier [11]. In emulsifier-free polymerization, an initiator [20] or water-soluble monomer [21] can act as a polymeric emulsifier. HEMA is a water-soluble monomer; however, polymers with HEMA units may not be stably dispersed in water easily. A high content of HEMA units or hydroxyl groups in a polymer favors the crosslinking reaction between HEMA units and amino resin [22], and this can guarantee high crosslinking density that enhances mechanical properties [18]. However, there exists difficulty in preparing an acrylic emulsion polymer with a high content of HEMA for the low stability of the emulsion, and the low stability is derived from crosslinked polymer led by the crosslinking, which is caused by transesterification among the pendant hydroxyl groups of the polymer chain [23,24] and the interpolymer complex. Our group identified coagulum over 0.5 wt.% and flocculation when an excessive amount of HEMA (≥25 wt.% total monomer mass) was added in the polymerization [25]. For this reason, the content of HEMA should not be too high when preparing a high-solid-content acrylic emulsion to restrain the crosslinked polymer. Thus, it is necessary to study a synthetic methodology to prepare a stable emulsion with HEMA units in the polymer chain. An interpolymer complex is formed in acidic conditions because of the hydrogen bonding between the hydroxyl and carboxyl groups in monomer units [26,27], and the complex decreases the stability of the emulsion. For this reason, the ammonium salt of MAA [28] was used as a polymeric surfactant to restrain the formation of the interpolymer complex in our previous paper, and a stable emulsion with a high content of hydroxyl functional monomer HEMA (19.4 wt.% of the total monomer mass) units was prepared; the highest *M*_n_ of the polymer could reach 34,100 g·mol^−1^, but the content of MAA in the total monomer mass was 12.3 wt.%. Furthermore, the emulsion viscosity with St units in the polymer chain was high, and the fluidity of the emulsion was not good. The carboxyl group in MAA units may corrode metal when ammonia solution is volatilized; hence, choosing a polymeric emulsifier that does not corrode metal and can stabilize an emulsion with St units in the polymer chain is necessary. Nonionic polymeric emulsifier PEGMA was added to stabilize the emulsion with St units [29,30,31], and the copolymer particles were uniform. In terms of the chemical structure, the group of poly(ethylene glycol) ethyl ether in PEGMA does not corrode metal. On the basis of the above understanding, PEGMA was chosen to study the preparation of a styrene–acrylic emulsion in this paper.

Emulsion polymerization can be conducted via controlled radical polymerization (CRP) [32]. In CRP, the controlled mechanism is based on the reversible deactivation of growing radicals [32,33]. An emulsion copolymer with PEGMA units and St units in the polymer chain can be prepared via CRP such as nitroxide-mediated polymerization (NMP) [21] and reversible addition fragmentation chain transfer polymerization (RAFT) [34,35,36]. The monomer benzyl methacrylate (BnMA) is analogous to St in chemical structure, and an emulsion copolymer containing PEGMA units and BnMA units can be prepared via photo-controlled iodine-mediated green reversible deactivation radical polymerization (RDRP) [37]. In our previous paper, an emulsion copolymer containing HEMA units and MAA units was prepared via RITP, and the emulsion was stable. Compared with NMP polymerization and RAFT polymerization, RITP does not require complicated chemicals to regulate the polymerization, and the chain transfer angents are synthesized in situ during the polymerization [38]. On the other hand, only a few of the chain transfer agents in RAFT are commercially available, and disadvantages of the polymer prepared by RAFT polymerization include its odor and color [39]. Iodine is commercially available, and there is no odor in the dried emulsion film. Thus, iodine transfer polymerization was chosen. Furthermore, PEGMA can be copolymerized with HEMA [24] or a monomer mixture comprising MAA and St [40], forming a stable emulsion. Therefore, it is probable that an emulsion copolymer containing PEGMA units, HEMA units, MAA units, and St units may be prepared via RITP emulsion polymerization.

**Figure 1:**
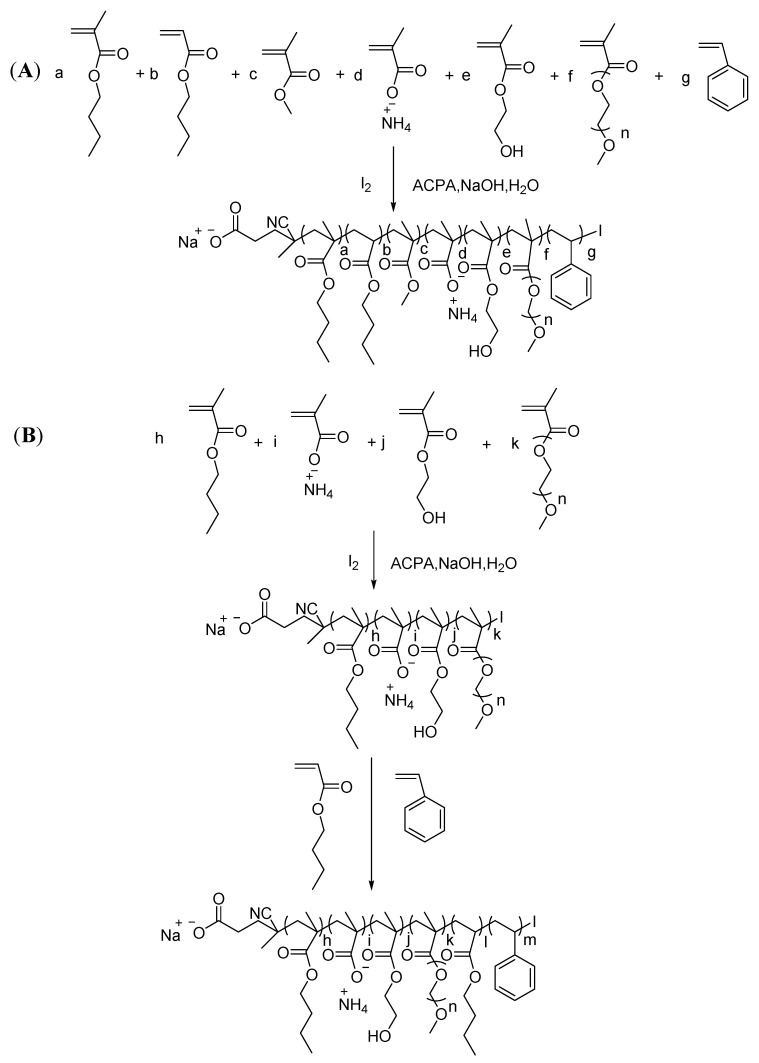
(**A**) Synthetic route of styrene–acrylic emulsion copolymer via RITP and (**B**) chain extension reaction via RITP.

In this paper, the copolymerization of St and an acrylate-based monomer (Figure 1) was studied via RITP emulsion polymerization. The research was conducted by changing the mass ratio of methyl methacrylate/St, the mass ratio of PEGMA/MAA, the amount of PEGMA, and the amount of iodine. Kinetics experiments of the random copolymerization and chain extension reaction with St units and BA units proved the living polymerization of the chain. The emulsion polymer was characterized by monomer conversion, viscosity, particle size, molecular weight, *T*_g_, particle morphology, and Fourier-transform infrared (FTIR) spectra. The protective properties of the modified film were measured by pencil hardness rank, adhesive property, toluene absorption, and water resistance. The mechanical properties of the dried emulsion film or modified film were measured by tensile experiment.

## 2. Materials and Methods

### 2.1. Materials

Ammonia solution (25–28 wt.%), *n*-butyl acrylate (BA), methyl methacrylate (MMA), styrene (St), *n*-butyl methacrylate (BMA), methacrylic acid (MAA), *p*-toluene sulfonic acid (TsOH), and sodium hydroxide (NaOH) were purchased from Sinopharm Chemical Reagent Co., Ltd. (Shanghai, China), as analytical reagent grade (AR). Methacrylic acid-β-hydroxyethyl ester (HEMA) was purchased from Tianjin Institute of Chemical Reagents, AR. Poly (ethylene glycol) methyl ether methacrylate (PEGMA, average molecular weight = 475 g·mol^−1^) was purchased from Shanghai Aladdin Biochemical Technology Co., Ltd (Shanghai, China). 4,4′–Azobis (4-cyanovaleric acid) (ACPA, 98%, AR), containing ca. 20% water, was purchased from Energy Chemical. *N*, *N*-Dimethylethanolamine (DMEA) was purchased from Tianjin Kemiou Chemical Reagent Co., Ltd. (Tianjin, China), AR. Hexamethylolmethymelamine (HMMM, MF Resin) was provided by H. J. Unkel Co., Ltd. (Zhuhai, China). The monomers BA, MMA, St, and BMA were extracted by washing four times with 10 wt.% aqueous sodium hydroxide solution in a separatory funnel, followed by washing with deionized water four times in a separatory funnel. Other materials were used as received.

### 2.2. Methods

#### 2.2.1. Emulsifier-Free Copolymerization of Acrylate Monomers

In a typical example illustrated in Figure 1A, BA (1.027 g, 8.01 mmol), BMA (6.162 g, 40.33 mmol), MMA (0.822 g, 8.21 mmol), St (1.233 g, 11.83 mmol), HEMA (2.618 g, 20.12 mmol), 9.138 g of deionized water, and 273 mg (1.074 mmol) of I_2_ were added to a flat-bottom flask (100 mL), and the mixture was stirred by a magnetic stirrer for 15 min. Thereafter, a neutralized MAA solution comprising MAA (1.664 g, 19.33 mmol), deionized water (4.141 g), and ammonia solution (1.49 g, 21.87–24.50 mmol NH_3_) was added to the flask and stirred for 15 min. Next, 3.461 g (1.235 mmol) of ACPA 10 wt.% solution (2.25 g ACPA, 642 mg NaOH, and 19.608 g H_2_O) was added. The reaction system was deoxygenated by bubbling with high-purity nitrogen for 25 min at room temperature while stirring. Afterward, the ingredients were placed under the atmosphere of high-purity nitrogen provided by a balloon, and the mixture was heated to 80 °C while stirring; the total heating time was 210 min. The reaction was ceased by exposure to air. The total theoretical monomer mass content by weight was 40 wt.%. The monomer conversion and the solid content were determined by gravimetric analysis.

When the mass ratio of the monomer or the mass of the I_2_ was changed, the procedures were as described above.

#### 2.2.2. Chain Extension Reaction with BA and St

Firstly, BMA (6.162 g, 40.33 mmol), HEMA (2.618 g, 20.12 mmol), deionized water (9.892 g), and I_2_ (164 mg, 0.644 mmol) were added to a flat-bottom flask (100 mL), and the mixture was stirred by a magnetic stirrer for 15 min. Thereafter, a PEGMA solution comprising PEGMA (0.832 g, 1.75 mmol) and H_2_O (2.27 g) was added into the flask and stirred for 15 min; then, a neutralized MAA solution comprising MAA (0.832 g, 9.66 mmol), deionized water (3.80 g), and ammonia solution (0.708 g, 10.39–11.64 mmol NH_3_) was added to the flask and stirred for 10 min. Afterward, 3.461 g (1.235 mmol) of ACPA 10 wt.% solution was added. The reaction system was deoxygenated by bubbling with high-purity nitrogen for 25 min at room temperature while stirring. Next, the ingredients were placed under the atmosphere of high-purity nitrogen provided by a balloon, and the mixture was heated to 80 °C while stirring. The sample was removed by a syringe under a nitrogen atmosphere after heating for 130 min (the first stage). Thereafter, the nitrogen gas-saturated mixture comprising BA (1.027 g, 8.01 mmol) and St (2.054 g, 19.72 mmol) was immediately added to the flask, and polymerization continued for 90 min (the second stage).

When the mixture comprising BA and HEMA was added at the second stage, the procedures were as described above. The reaction time of the first stage was 168 min, and that of the second stage was 33 min.

#### 2.2.3. Modification of the Emulsion Polymer

Firstly, the emulsion (2.5 g) and deionized water (2.08 g) were added to a round flask (25 mL) at room temperature, and then stirred for 5 min. Afterward, HMMM (0.64 g) was added while stirring for 10 min. TsOH solution (85 µL, 10 wt.%) was added dropwise to the mixture, and the mixture was stirred for 1 h.

To measure the hardness, adhesive properties, and water resistance, the ingredients of the modified film were coated on a clean tinplate and coverslip, and these samples were placed in room temperature for 2 h to evaporate water naturally. Thereafter, the tinplate and coverslip were heated at 80 °C for 120 min and then 150 °C for 40 min. After heating, the tinplate and coverslip were cooled to room temperature via natural cooling.

To measure tensile strength, the ingredients of the modified film were coated on a horizontal tetrafluoroethylene plate, and the samples were placed in room temperature for 2 h to evaporate water naturally. The plate was heated at 80 °C for 120 min and then 150 °C for 40 min. Afterward, the modified film could be torn off the plate.

#### 2.2.4. Preparation of Dried Emulsion Films

To measure tensile strength, the emulsion mixture was poured onto a horizontal tetrafluoroethylene plate, and the emulsion was dried under the radiation of an infrared lamp for at least 24 h; then, the film could be torn off the plate. At least three samples of the layer could be obtained per piece of emulsion film.

### 2.3. Characterizations

#### 2.3.1. Viscosity

The emulsion viscosity was measured using a DV-79 digital viscometer (Shanghai Ni Run Intelligent Technology Co., Ltd., Shanghai, China) with a rotor (E-type rotor, F-type rotor, or G-type rotor) at 25 °C. The rotational rate of the rotor was 75 or 750 rpm when the viscosity was in the corresponding measurement range of the rotor type and rotational rate.

#### 2.3.2. Monomer Conversion

The determination of monomer conversion was as follows:

(1) Conversion%=m (Dried emulsion)/(m (Emulsion solution)×w (Total monomer))

where w (Total monomer) is the total mass content of the monomer in weight.

#### 2.3.3. Particle Diameter

After adding deionized water to the emulsion according to the volume, the emulsion sample was diluted 1000-fold. Then, the particle size diameter and the particle size distribution of the diluted emulsion sample were measured using a Zetasizer Nano ZS laser particle sizer, Malvern Instruments Ltd. (Shanghai, China) at 25 °C; the Malvern Zetasizer ZS device that we used worked at 173° in backscattering mode.

#### 2.3.4. Molecular Weight

The emulsion sample was further neutralized using DMEA, and the excess DMEA could be removed via rotary evaporation under reduced pressure. Afterward, the sample was dispersed in THF (20 mg/mL), and the mixture was filtered using an organic phase filter head. Then, 50 µL of the filtered sample was injected into the device. The number average molecular weight (*M*_n_), the weight average molecular weight (*M*_w_), and the index of the molecular weight distribution (*Ð*) of the polymer were measured using a gel permeation chromatograph (GPC) from US WATERS Corporation (Milford, MA, USA). The GPC device was equipped with a Waters-2414 Refractive Index Detector, and the eluent THF flowed at a rate of 1.0 mL/·min at 25 °C. The material of the GPC columns was styrene–divinylbenzene copolymer. The GPC columns used were WATERS Styragel HR1 + Styragel HR3 + Styragel HR4. The calibrated material in the chromatographic column was polymethyl methacrylate.

#### 2.3.5. Glass Transition Temperature

*T*_g_ was tested using a TA Q20 DSC Instrument (TA Instruments, New Castle, DE, USA), and the measurement was conducted under nitrogen atmosphere. The measuring temperature range was −15 °C to 120 °C, and the heating/cooling rate was 10 °C/min.

#### 2.3.6. Fourier-Transform Infrared Spectroscopy

The sample was prepared by drying the emulsion under the radiation of an infrared lamp. The infrared spectrum of the sample was tested using a Thermo Nicolet Fourier-transform infrared spectroscope (FTIR) (Thermo Fisher, Waltham, MA, USA), and the device was equipped with a diamond from Smiths Detection for attenuated total reflectance (ATR). The scanning range was 4000–500 cm^−1^.

#### 2.3.7. Transmission Electron Microscopy

The 1000-fold diluted sample was added to the copper mesh, and then stained with 3% phosphotungstic acid (PTA) solution. After evaporation of water in the mesh, the mesh was observed using a JEM-2100 transmission electron microscope from JEOL Corporation (Tokyo, Japan), with an accelerating voltage of 220 kV. The sample morphology could be observed after vacuuming.

#### 2.3.8. Hardness Rank of the Modified Film

The hardness of the modified film was tested by sliding pencil lead on the film surface. The procedures and the hardness rank were conducted according to China National Standard GB/T 6739-2006/ISO 15184:1998.

First, the pencil was pressed down on the film surface at an angle of 45° by hand. The pencil was shifted at a rate of 0.5–1 mm/s and for at least 7 mm. If the length of the marking was longer than 3 mm, a pencil with lower hardness was used until no marking longer than 3 mm existed. When a marking shorter than 3 mm existed, the pencil hardness rank was regarded as the hardness rank of the film. This measurement was conducted at a temperature of 23 ± 2 °C. The measurement was conducted at least three times.

#### 2.3.9. Adhesive Property Rank of the Modified Film

The adhesive property was evaluated by the affected area of cuts on the surface of the modified film or dried emulsion film adhered to the tinplate piece. The procedures and the evaluation standard were conducted according to China National Standard GB/T 9286-1998 equivalent ISO 2409:1992.

Six cuts were made in each direction of the lattice pattern, and the lattice was made by scratching the knife under the surface of the film with the use of ruler. The spacing of the cuts in each direction were equal to 1 mm (0–60 μm thick film) or 2 mm (60–120 μm thick film). The tape was removed on the surface of the cuts by grasping the free end and pulling it off in 0.5–1.0 s at about 60°. When none of the lattice squares were detached, classification was regarded as rank 0. When 0–5%, 5–15%, 15–35%, and 35–65% of the area was affected, classification was regarded as 1, 2, 3, and 4, respectively. Any degree of flaking that could not be classified as 4 was classified as 5. This measurement was conducted at a temperature of 23 ± 2 °C. The measurement was conducted at least three times.

#### 2.3.10. Water Resistance of the Modified Film

The water resistance of the modified film was measured by the change in the film immersed in boiling water. The test was based on a comparison with a modified film not immersed in boiling water. The procedures and the evaluation standard were conducted according to China National Standard GB/T 1733-1993.

Water resistance of the films was determined by immersing three sample pieces into boiling water for 2 h, and then the immersed part was dried by filter paper. Next, the appearance of these three sample pieces in terms of gloss, whitening, wrinkling, etc. was compared with those not immersed in boiling water.

#### 2.3.11. Water Absorption of the Modified Film

The modified film was weighed. Then, the same piece of modified film sample was immersed in deionized water for 24 h. After 24 h, the sample immersed in water was weighed. The determination of water absorption was as follows:

Water absorption% = 100% × m (Dried film)/(m (Film immersed in water)).(2)

#### 2.3.12. Toluene Absorption of the Modified Film

The modified film was immersed in toluene for 24 h. The determination of toluene absorption was as follows:

Toluene absorption% = 100% × m (Dried film)/(m (Film immersed in Toluene))(3)

#### 2.3.13. Tensile Strength

Tensile strength of the dried emulsion film or modified film was measured using an Electronic Universal Testing Machine from MTS SYSTEMS (China) Co., Ltd. (Shanghai, China) with SANS-Power Test software, and the measurement was conducted at 10 mm·min^−1^ at 20 ± 2 °C. The shape of the film was rectangular in general. The length of the film was longer than 12 mm, and the width ranged from 5–12 mm.

## 3. Results and Discussion

### 3.1. Participation of Styrene in Emulsifier-Free Polymerization

As shown in Table 1, when all hard monomer MMA was replaced by St, the emulsion was not stable throughout the reaction when only the ammonium salt of MAA was used as a polymeric emulsifier in the emulsion system. In Table 1 and Table 2, the total mass of MMA and St was constant, while the mass ratio of MMA/St was changed; the mass of other ingredients was constant. When hard monomer MMA participated in the polymerization, the emulsion was stable, the monomer conversion was higher than 95%, and the solid content was over 40 wt.%. The monomer conversion was not changed obviously for the four mass ratios. The emulsion was stored and remained stable for 6 months. The emulsion polymer had a measured *M*_n_ range from 23,000 to 30,000 g·mol^−1^, which is analogous to the range in our previous paper. The measured *M*_n_ was higher than the theoretical *M*_n_. Iodine is hydrolyzed in water [41], and this hydrolysis decreases the amount of iodine that is involved in the synthesis of the chain transfer agent in situ [42,43,44]. Furthermore, ammonia solution facilitates hydrolysis. Thus, the deviation of measured *M*_n_ from the theoretical *M*_n_ was caused by the hydrolysis of I_2_ in water. The measured *M*_n_ increased with the increase in MMA. The index of the molecular weight distribution decreased overall with the increase in MMA. According to the work of Tonnar et al. [38,45], the index of the molecular weight distribution with iodine in the polymerization was smaller than that without iodine, and the index with iodine in the polymerization ranged from 1.40 to 2.20 in most cases. In comparison with typical controlled radical emulsion polymerization such as RAFT polymerization [8,36] or atom transfer radical polymerization (ATRP) [46], the index of the molecular weight distribution in RITP was relatively large. However, the diameter of the particle (*d*_p_) was not increased with the increase in MMA, and the *d*_p_ ranged from 350–430 nm. When the mass ratio of MMA to St was 4:6, the viscosity value of emulsion was 2350 mPa·s at 750 rpm. This viscosity was larger than that (525 mPa·s at 750 rpm) in our previous paper with the mass ratio of BA to MMA kept at 1:2 [28]. More MMA led to an overall decrease in the emulsion viscosity. Side products are unavoidable because chain transfer occurs due to collision between two active centers attached to polymer chains in polymerization [47] or emulsion polymerization [48,49,50]. Experimentally, an emulsion with lower viscosity can improve the diffusion of the added hydrophobic monomer or monomer mixture, and this may ensure that random copolymerization or block copolymerization with St monomer units is conducted more fluently. The results in Table 1 show that, when all MMA was replaced by St, the emulsion was not conducted fluently with only the ammonium salt of MAA used as a polymeric emulsifier, whereas when both St and MMA were present, the emulsion was stable and of low viscosity.

Before modification by MF resin, the pencil hardness rank of the three kinds of dried emulsion film was 1 H in room temperature, and their adhesive property rank was 1. Furthermore, the dried film was translucent, and it could be dispersed in water when the mixture was stirred. The cured film surface became hard with a pencil hardness rank of 2 after the modification in Table 2. The reason is that a crosslinked structure polymer was prepared via the reaction of hydroxy functional acrylics with MF resin [22], and this crosslinked structure limited the movement of the segment [18], thus leading to the hardness of the polymer film being higher than that of the dried emulsion film. Furthermore, the adhesiveness rank of the cured film was 0, suggesting that the adhesive property of the modified film was better than that of the dried emulsion film. Toluene absorption at the mass ratio of 10/0 was higher than that for the other three mass ratios; hence, the toluene resistance of the cured film was increased when St was added. The cured film was not dissolved in toluene, as polymers with a crosslinked structure only swell in some kinds of organic solvent [51]. Water absorption decreased when the mass ratio of MMA/St increased. The cured film was translucent after immersing in boiled water when the monomer mass ratio was 6/4 or 10/0, indicating water resistance. The best results in terms of good pencil hardness, good adhesive property, low toluene absorption, low water absorption, and good water resistance of the modified film were obtained when the mass ratio of MMA/St was 6/4.

Styrene is a hydrophobic monomer, and the phenyl structure of St units in the polymer promotes chain rigidity. Poly (ethylene oxide) (PEO) in the pendant group of a polymeric emulsifier can form a dense protective layer around the surface of the latex particles [29,52] prepared via conventional radical emulsion polymerization. Some reports have proposed that hydrophilic polymeric emulsifiers such as poly (ethylene glycol) ethyl ether methacrylate (PEG-EEA) can be copolymerized with St to prepare a stable emulsion [29,30,53] via conventional radical polymerization. Furthermore, MAA can cooperate with PEGMA to stabilize an emulsion with St units [40] via conventional radical polymerization. Considering the above properties of polymers containing St monomer units, a high-solid-content styrene–acrylic emulsion may be prepared by adding PEGMA to an RITP system. As shown in Table 3, when there was no PEGMA in the polymerization system, the emulsion was of high viscosity and poor fluidity. In Table 3 and Table 4, the total mass of PEGMA and MAA was constant, while the mass ratio of PEGMA/MAA was changed; no MMA was added, and St was added; the mass of other ingredients was constant. Increasing the mass content of PEGMA resulted in lower monomer conversion, lower molecular weight, higher index of molecular weight distribution (*Ð*), lower diameter, lower viscosity, and good fluidity. The dense protective layer derived from PEGMA on the latex particles may hinder the access of hydrophobic monomers such as St, BA, and BMA, which may not be beneficial for the increase in particle size. The steric effects depend upon the size of the substituents [54], whereby larger substituents in the PEGMA may lead to a larger steric effect; thus, propagating chain radicals with PEGMA leads to a larger steric effect than that with ammonium salt of MAA, resulting in lower reactivity, a lower propagating reaction rate, and lower monomer consumption. Therefore, an increase in PEGMA may also lead to a lower molecular weight and lower monomer conversion. Furthermore, the dense protective layer may decrease the frictional effect between particles, which may decrease the viscosity of the emulsion. The emulsion was stable when the mass ratio of PEGMA/MAA was 5/5, indicating that half the referenced mass amount of MAA could be used to prepare a stable emulsion with analogous *M*_n_ range and low viscosity. However, when the mass ratio of PEGMA/MAA was 7/3, the stability of the emulsion was not good, and a little white precipitate existed in the emulsion. This phenomenon indicated that enough MAA is needed to guarantee higher monomer conversion and maintain the stability of the emulsion with St units in the polymer chain. Furthermore, a high mass content of PEGMA may not guarantee high monomer conversion, high molecular weight, and low polydispersity index in this polymerization system. The above results show that the cooperation of PEGMA and MAA facilitated stabilization of the styrene–acrylic emulsion, and all MMA monomers could be replaced by St.

From the perspective of the chemical structure of PEGMA, no groups in PEGMA monomer units exist that can react with MF resin. This property is different from that of MAA units or HEMA units. Thus, it is necessary to research the influence of the mass ratio of MAA/PEGMA on the properties of the cured film. The pencil hardness rank of the cured film was not changed by the mass ratio of MAA/PEGMA, as shown in Table 4. The adhesive property worsened when the mass ratio of PEGMA/MAA was 7/3. The water absorption decreased with the increase in mass ratio, which may be because less MAA decreased the hydrophilicity of the chain segment in the crosslinked film when no MMA participated in polymerization. The best results in terms of good pencil hardness, good adhesive property, and low water absorption were obtained when the mass amount of PEGMA was equal to that of MAA.

From the perspective of the chemical structure of PEGMA, it can be used as a polymeric emulsifier. However, the emulsification ability of PEGMA in this emulsion polymerization was unknown. As shown in Table 5, the emulsion was stable when the polymeric emulsifier consisted of neutralized MAA, and all St monomer units were replaced by MMA. However, the emulsion was not stable when all MAA was replaced by PEGMA, and there existed flocculation that could not be dispersed in water or THF. These phenomena suggest that the emulsification ability of neutralized MAA units in the polymer chain was stronger than that of PEGMA, and the emulsion was not stable when all neutralized MAA was replaced by PEGMA. The hydrophilic/lipophilic balance (HLB) value of the ammonium salt of MAA is 21.25, while that of PEGMA is 9.68. Thus, the lipophilic property of PEGMA is stronger than that of neutralized MAA. However, the steric hindrance due to the side group of PEGMA is larger than that of neutralized MAA, which may result in a lower reactivity of propagating radical with PEGMA in the chain than that of the neutralized MAA. In summary, the emulsion ability of neutralized MAA throughout the polymerization period was stronger than that of PEGMA. St is more hydrophobic than MMA; hence, the emulsion polymerization with St units must be conducted with the addition of neutralized MAA.

As shown in Table 3, when the mass ratio of PEGMA/MAA was 3/7, the viscosity was much higher than that of the others. As mentioned in Table 1, changing the mass ratio of MMA/St could tune the viscosity when no PEGMA units existed in the polymer chain. Similarly, changing the mass ratio of MMA/St could tune the viscosity when MAA and PEGMA were used as polymeric emulsifiers. This experiment was done, and the results are shown in Table 6. In Table 6 and Table 7, the total mass of MMA and St was constant, while the mass ratio of MMA/St was changed; the mass of other ingredients was constant, and the monomer conversion was higher than 95%. The monomer conversion decreased when the mass ratio was increased from 0/10 to 8/2. The measured molecular weight decreased when the mass ratio of MMA/St was increased from 0/10 to 4/6, while the measured molecular weight was reduced when the mass ratio of MMA/St was increased from 4/6 to 8/2. The measured molecular weight ranged between 21,000 and 28,000 g·mol^−1^, and the index of molecular weight distribution ranged between 1.55 and 1.87. The largest *d*_p_ existed when the mass ratio of MMA/St was 8/2, and *d*_p_ at the six mass ratios ranged from 330 to 430 nm. When no MMA or no St took part in the polymerization, the viscosity was higher than 1100 mPa·s. The smallest viscosity existed when the mass ratio of MMA/St was 4/6. The viscosity of the emulsion with no St units in the polymer chain was larger than that with both St and MMA units in the polymer chain. This indicates that a styrene–acrylic polymer with analogous molecular weight and lower viscosity could be prepared when MAA was combined with PEGMA, and the emulsion with some MMA replaced by St was stable when PEGMA was added to the system.

In Table 7, the properties of the cured film synthesized by emulsion with MMA units and St units in the polymer chain are shown. Here, the pencil hardness of the cured films was at rank 2 in most cases, and the adhesive property was good for all six mass ratios of MMA/St. The toluene absorption of the cured film ranged between 12.3 wt.% and 16.1 wt.%, and the difference was overall minimal. The water absorption of the cured film ranged between 2.20 wt.% and 5.75 wt.%. The best results in terms of good pencil hardness, good adhesive property, low toluene absorption, low water absorption, and good water resistance of the modified film were obtained when the mass ratio of MMA/St was 10/0.

In summary, PEGMA could be combined with MAA in the reaction system to stabilize a styrene–acrylic emulsion with HEMA units in the polymer chain, and an emulsion polymer with analogous molecular weight range and relative low viscosity was synthesized. Moreover, the cured film exhibited a good hardness property, good adhesive property, low toluene absorption, low water absorption, and good water resistance.

### 3.2. Influence of the Amount of PEGMA on Emulsifier-Free Polymerization

In our previous paper, MAA accounting for at least 8.6 wt.% of the total monomer mass was added to stabilize the emulsion; however, the viscosity of the polymerization upon changing the amount of MAA was higher than 1200 mPa·s for most of the experiment [28]. As mentioned before, the viscosity of the emulsion without MMA units in the polymer chain could be decreased when the proportion of PEGMA was increased. Furthermore, the emulsion with St units in the polymer chain was stable and of relatively low viscosity for the combination of MAA and PEGMA. Therefore, it is necessary to study the influence of the amount of PEGMA on the emulsion when both MMA and St are involved in the polymerization.

In Table 8 and Table 9, the mass of PEGDMA was changed, and the mass ratio of MMA/St was constant; the mass of other ingredients was constant, and the total mass content was constant. Monomer conversion was over 98%, and the solid content of the emulsion could reach 45 wt.%, as shown in Table 8. The monomer conversion did not change obviously with the change in mass ratio. The measured molecular weight decreased when the mass ratio PEGMA/MAA was increased from 1/7 to 5/7. The reason may be that PEGMA could be used as an emulsifier and monomer, and the polymeric emulsifier favored the generation of oligomers, whereas the generated oligomers increased the index of molecular weight distribution (*Ð*) and the PDI; *Ð* or PDI was increased at this mass ratio. The molecular weight was increased when the mass ratio was increased from 5/7 to 12/7. The reason may be that PEGMA was used as a monomer, and the concentration of monomers increased with the increase in PEGMA amount, while the increase in monomer concentration could increase the average degree of emulsion polymerization [55]. The measured molecular weight ranged between 18,000 and 22,000 g·mol^−1^, and the index of molecular weight distribution ranged between 1.70 and 1.91. Larger-diameter particles existed when the mass ratio of PEGMA/MAA was 5/7 or 7/7. The viscosity ranged from 520 mPa·s to 770 mPa·s in most cases. When the mass ratio of PEGMA/MAA was between 5/7 and 12/7, the viscosity was increased with the increase in PEGMA, but the tendency was opposite for the molecular weight distribution index and the diameter. The emulsion was stable throughout the reaction and remained stable for 6 months.

**Figure 2:**
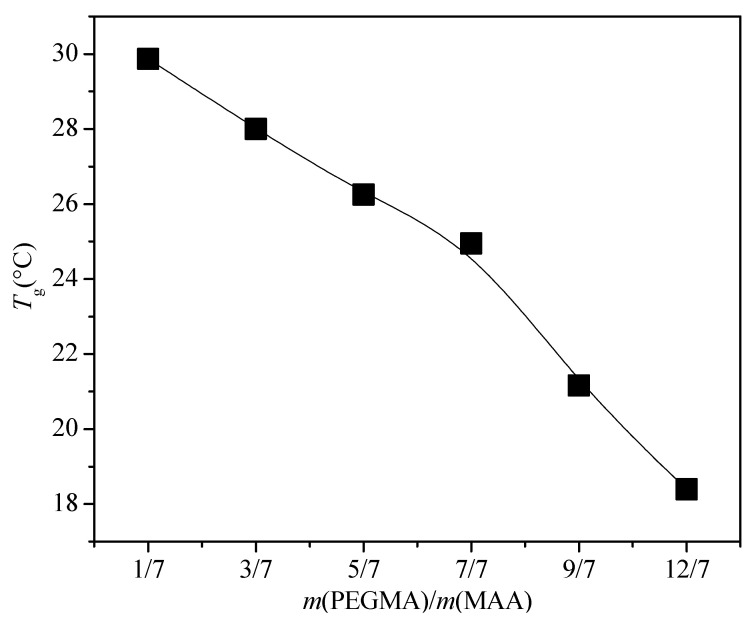
*T*_g_ of the emulsion polymer with different mass ratios of PEGMA/MAA.

The *T*_g_ of the emulsion polymer decreased with the increase in PEGMA, as shown in Figure 2. According to the Fox equation of *T*_g_ [14], *T*_g_ of a random copolymer can be changed as a function of the weight fractions of the monomer unit and *T*_g_ of the component monomer, whereby more of the soft monomer unit leads to a decrease in the polymer *T*_g_. The *T*_g_ of the PEGMA homopolymer (*M*_n_ of PEGMA monomer is 475 g·mol^−1^) is −62.8 °C [56]; hence, PEGMA is a soft monomer. Thus, a greater amount of PEGMA would lead to a decrease in the *T*_g_ of a random polymer. Moreover, the *T_g_* at all six mass ratios was in the range of room temperature applied for acrylic resin, indicating that the emulsion has a potential application in coating.

As illustrated in Table 9, the pencil hardness rank of the cured film was at 2 H in most cases, and the adhesive property was good for all six mass ratios of PEGMA/MAA. The hardness and the adhesive property were not influenced by the mass ratio of PEGMA/MAA. Water absorption ranged between 2.80 wt.% and 7.90 wt.%. Water absorption was increased between the mass ratios of 1/7 and 7/7. The reason may be that, when the amount of MAA was constant and the amount of PEGMA was less than MAA, more PEGMA was located on the surface of the film, as PEGMA units are hydrophilic. PEGMA is a soft monomer, and PEGMA units favor the movement of chain segments in the crosslinking reaction, thereby allowing more polymer to react with amino resin. This may have enhanced the crosslinking reaction and decreased the hydrophilicity of the modified film. Thus, water absorption was decreased between the mass ratios of 7/7 and 12/7.

**Figure 3:**
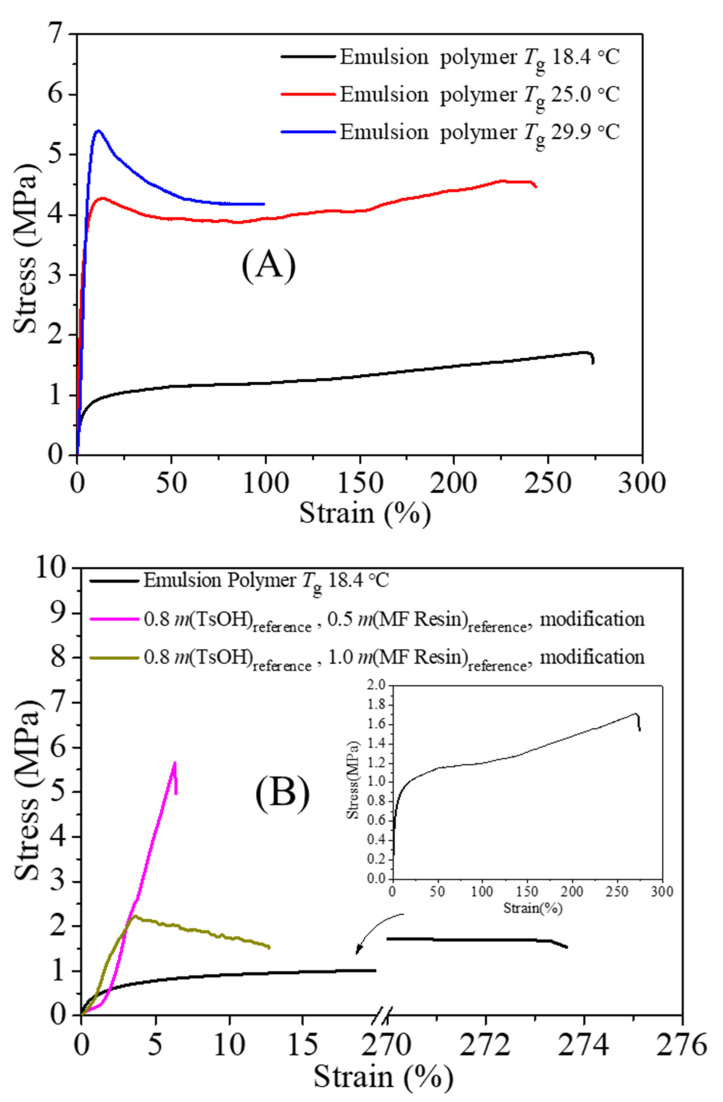
Stress–strain curves of (**A**) dried emulsion polymer film, and (**B**) the film modified via reaction of emulsion polymer with MF resin.

The tensile strength and the elongation of dried emulsion polymer films are shown in Figure 3. In Figure 3A, the dried emulsion polymer film with *T*_g_ = 29.9 °C exhibited the largest maximum tensile strength (over 5.0 MPa). The maximum tensile strength of the dried emulsion polymer film was increased when the *T*_g_ of the emulsion polymer was increased, but the trend was opposite for the elongation at break. Below *T*_g_, there is insufficient energy for whole segments of the polymer chains to move; hence, the polymer film is stiff and deformation is resisted [57]. When the film is elongated at a temperature lower than *T*_g_, a higher *T*_g_ of the tested polymer leads to higher energy, enabling whole segments to move and more outside force to elongate the polymer film. Thus, the maximum tensile strength of the dried emulsion film increased with the increase in *T*_g_ when the tested temperature was below *T*_g_. The largest maximum tensile strength (5.39 MPa) in the polymer with *T*_g_ = 29.9 °C was higher than that of the polyacrylate polymer with *T*_g_ = 43.9 °C (2.98 MPa) in our previous paper, indicating that the styrene–acrylic emulsion has potential application in preparing materials with mechanical properties.

The maximum tensile strength of the modified film was higher than that of the film unmodified, as shown in Figure 3B. When half the mass of the reference MF resin was used, the maximum tensile strength of the modified film was more than 5.5 MPa. These results indicate that the tensile strength property of the emulsion film could be improved via modification of the polymer with HEMA units. Hydroxyl groups from HEMA units in the polymer chain react with MF resins to form a crosslinking structure [22], which restricts the motion of the polymer chains [18]; therefore, the strength of the modified film was higher than that of the emulsion film without modification.

In conclusion, the best results in terms of measured molecular weight, index of molecular weight distribution, particle size, viscosity, solid content, adhesive property rank, pencil hardness rank, and maximum tensile strength were obtained when the mass ratio of PEGMA/MAA was 7/7.

### 3.3. Influence of Iodine on Copolymerization

In Table 10, no MMA was added, hard monomer St was added, and the mass ratio of PEGMA/MAA was 1/1; the mass of iodine was changed. As shown for runs 1a to 5a in Table 10, the molar ratio of ACPA/I_2_ was 1.15 when 1.0 times the mass amount of ACPA was added, and the measured *M*_n_ increased overall with the decrease in iodine, while the highest measured *M*_n_ was no more than 30,000 g·mol^−1^. However, the increment of *M*_n_ was not significant. The monomer conversion was no more than 97%, and the solid content was no more than 41 wt.%. In Tonnar’s work [38], the molar ratio of ACPA/I_2_ was 1.6, and the measured *M*_n_ increased obviously with the decrease in I_2_ in the presence of ACPA, while the highest measured *M*_n_ was 47,000 g·mol^−1^. This molar ratio of ACPA/I_2_ could be used to prepare a polymer with a measured *M*_n_ of more than 30,000 g·mol^−1^. When 1.4 times the mass amount of ACPA was added, the molar ratio of ACPA/I_2_ was 1.61, the measured *M*_n_ was increased from 19,400 g·mol^−1^ to 32,900 g·mol^−1^ (as shown for runs 1b to 5b in Table 10), and the largest measured *M*_n_ was more than 30,000 g·mol^−1^. As shown for runs 3a to 3b or runs 5a to 5b, the measured *M*_n_ for 1.4 times the reference mass amount of ACPA was higher than that for 1.0 times the reference mass amount of ACPA; the reason for this phenomenon is unknown. The monomer conversion was more than 98%, and the solid content was over 42 wt.%. The monomer conversion in Table 10 did not change obviously overall when the iodine amount was increased. The monomer conversion, diameter, and solid content for 1.4 times the reference mass amount of ACPA were higher than those for 1.0 times the reference mass amount of ACPA.

**Figure 4:**
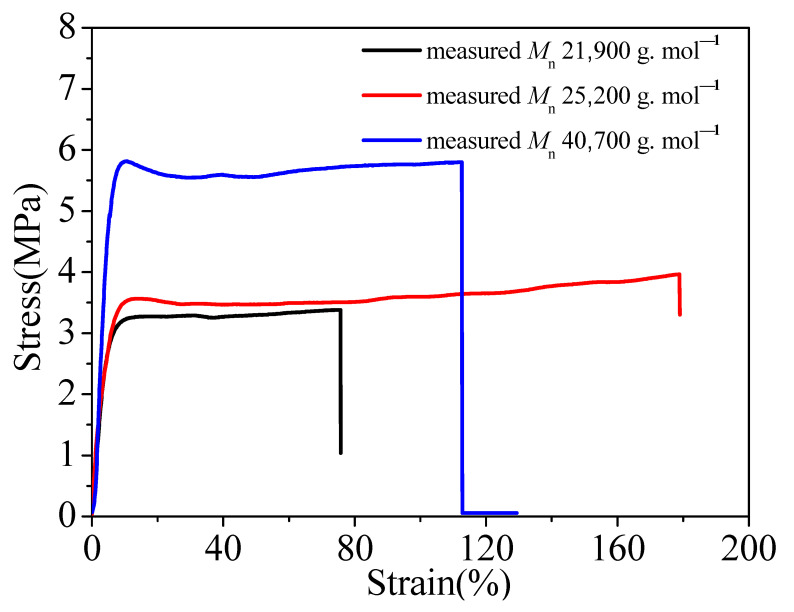
Stress–strain curves of emulsion film with different measured molecular weights.

The hard monomer St is usually combined with MMA in radical polymerization for determination of the copolymerization parameters based on kinetic data and quantum-chemical considerations [58]. Thus, we investigated whether the above molecular weight tendency in Table 10 existed in the styrene–methyl methacrylate-based styrene–acrylic emulsion polymer. In Table 11, the mass ratio of MMA/St was 4/6, the mass ratio of PEGMA/MAA was 3/7, and the mass of iodine was changed. As shown for runs 1b to 3b in Table 11, the monomer conversion was not changed obviously with the increase in the iodine amount, but the measured *M*_n_ for 1.4 times the mass amount of ACPA increased obviously with the decrease in iodine, and the highest measured *M*_n_ could reach 40,000 g·mol^−1^. This molecular weight tendency could have led to some changes in the mechanical property of the polymer film. Thus, it was necessary to measure the tensile strength of the dried emulsion film with different measured *M*_n_. The maximum tensile strength was increased with the increase in polymer *M*_n_, and the largest maximum tensile strength was more than 5.5 MPa, as shown in Figure 4. Polymer chains with a high molecular weight become large and are, hence, entangled [18]; thus, a higher molecular weight promotes entanglements, which can act as junction points and govern the material’s mechanical response [59]. Thus, a polymer with a high molecular weight exhibits high strength, including tensile strength. The elongation at break of the dried emulsion film with *M*_n_ 40,700 g·mol^−1^ was more than 100%, indicating flexibility of the polymer film. Therefore, the polymer with the highest *M*_n_ over 40,000 g·mol^−1^ has some significance, and this polymerization methodology may provide potential application for preparing styrene–acrylic emulsions used in materials with excellent mechanical properties.

The above result indicates that a styrene–acrylic emulsion polymer with a relatively higher molecular weight could be prepared by reducing the I_2_ amount when 1.4 times the mass amount of initiator was used, and the dried emulsion film could exhibit a larger maximum tensile strength at higher molecular weight. Therefore, the polymer film has potential application in materials with excellent mechanical properties.

### 3.4. Kineticks in RITP Emulsion Copolymerization

**Figure 5:**
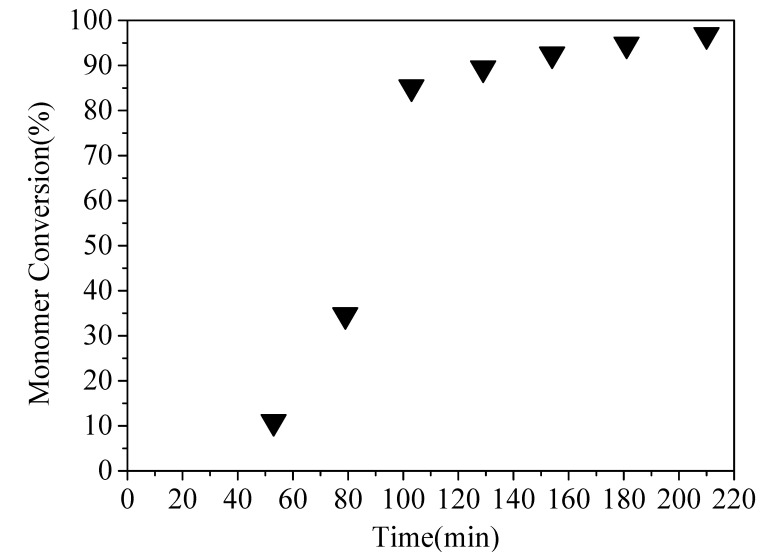
Evolution of monomer conversion versus time for RITP emulsion copolymerization. Conditions: *m*(PEGMA)/*m*(MAA) = 1/1 and *m*(MAA) + *m*(PEGMA) = 1.664 g; *m*(BA)/m(St) = 1/2; *n*(St)/*n*(HEMA)/*n*(BMA)/*n*(BA)/*n*(ACPA)/*n*(I_2_) = 18.36/18.74/40.36/7.46/1.15/1, ammonia solution (0.70 g); *m*(I_2_) = 0.273 g; *m*(ACPA) = 0.346 g; no MMA in the polymerization system; the total mass of ingredients without ACPA Solution and I_2_ was maintained at 30.08 g in theory; the reaction time was 210 min.

The monomer conversion was increased with time after 40 min, as shown in Figure 5. In the first 40 min of reaction time, the mixture was brown, indicating that iodine was not completely consumed in the induction period.

A sample was withdrawn using a syringe under N_2_ atmosphere at 53 min, and the mixture solution was brown, indicating that the induction period was more than 53 min. The polymerization was not complete when the monomer conversion was low, and there were odors coming from monomers in the emulsion. Subsequently, a sample was withdrawn at 79 min, and the emulsion was stable and white. This manifests that iodine was completely consumed in 79 min, with the sample exhibiting a certain viscosity. The monomer conversion did not increase obviously after 180 min. The highest monomer conversion was 96.8%. This tendency of the monomer conversion versus time in Figure 5 is similar to that reported in the work by Tonnar [38].

**Figure 6:**
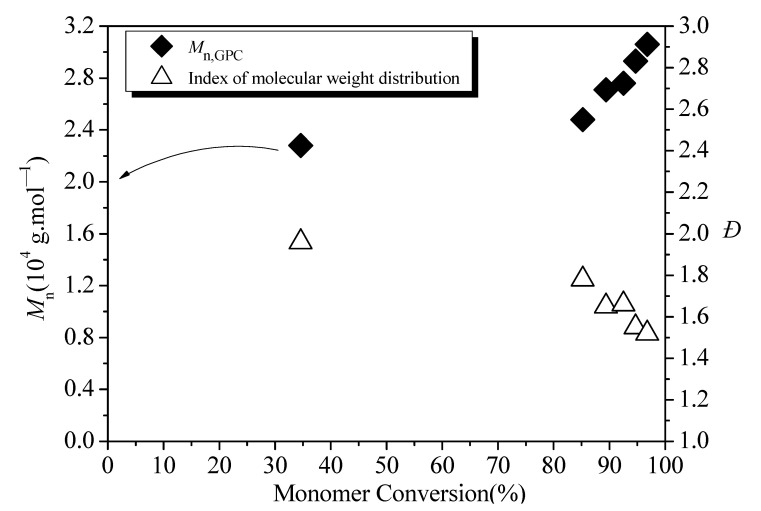
Evolution of measured *M*_n_ and *Ð* with monomer conversion for RITP emulsion copolymerization.

As shown in Figure 6, the evolution of measured *M*_n_ versus monomer conversion showed a linear trend, and *Ð* was decreased overall with conversion. The measured *M*_n_ in the withdrawn sample at 79 min (34.6% monomer conversion) was 22,800 g·mol^−1^, and this value deviated from the trend of the other five *M*_n_ values. The overall trend of *M*_n_ versus monomer conversion indicated a living polymerization process.

### 3.5. Chain Extension Reaction with St and BA in Emulsion Polymerization

A random copolymer was prepared, and monomer conversion was over 99.5%, as shown in Table 12. The emulsion of the random copolymer was stable and milky white with a weak blue color. After the first polymerization stage, the monomer mixture comprising water-insoluble monomer BA and St was added at the second polymerization stage. The emulsion at the end of the second polymerization stage was stable and milky white with a weak blue color, indicating that the random copolymer poly (PEGMA-*co*-MAA^a^-*co*-HEMA-*co*-BMA) can be used as a macro-emulsifier in the chain extension reaction. The measured *M*_n_ of the copolymer at the second stage was higher than that of the random copolymer at the first polymerization stage, indicating the living polymerization of the seed polymer chain. The molecular weight distribution index (*Ð*) at the second stage was lower than that at the first stage. In Tonnar’s work [38,60], *Ð* was decreased after the chain extension reaction in emulsion polymerization, and these results further indicate living RITP. Therefore, the molecular weight distribution decreased after the chain extension reaction in this paper, proving living polymerization. The diameter of the seed polymer was lower than that of the block copolymer, due to the growth of the seed polymer chain in the chain extension reaction. The solid content of the copolymer at the second stage was higher than that of the random copolymer at the first polymerization stage due to the addition of monomer mixtures at the second stage. These results indicate that the random copolymer poly (PEGMA-*co*-MAA^a^-*co*-HEMA-*co*-BMA) can be used as macro-chain transfer agent to control the chain extension reaction containing water-insoluble monomer BA and St in this polymerization system, and the seed random copolymer exhibited living polymerization.

**Figure 7:**
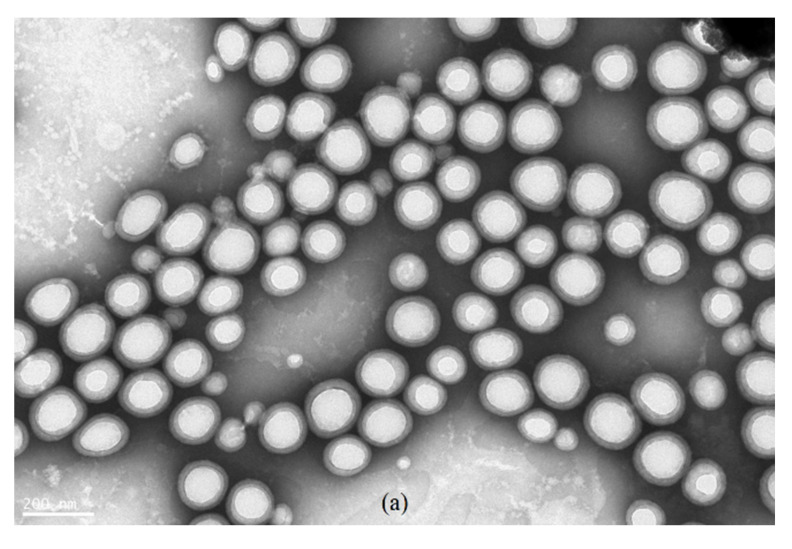

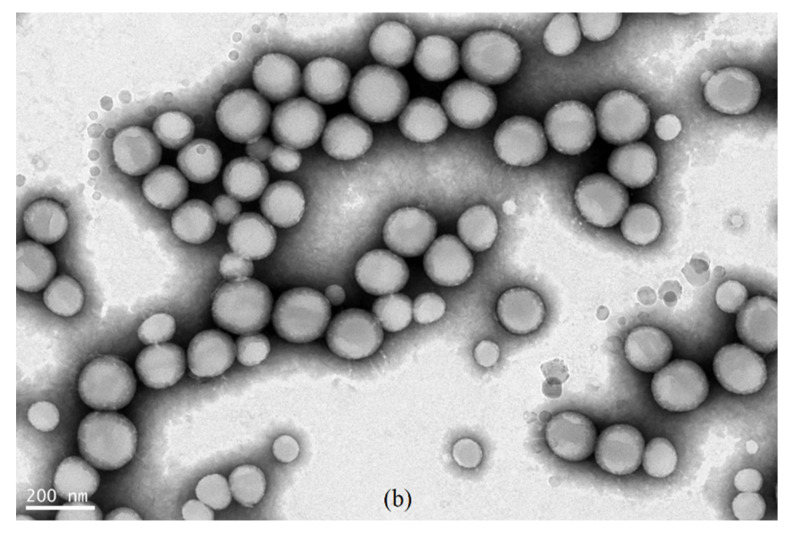
TEM micrograph of (**a**) the seed polymer and (**b**) the block copolymer described in Table 12.

As shown in Figure 7, the core–shell microstructure of the random copolymer was obvious. The core microstructure of the block copolymer was vague, and the shell layer was relatively thinner than that of the random copolymer. The shape of both random copolymer and block copolymer micelles was regular, and the core layer was completely covered by the shell layer. The diameter of the block copolymer in Figure 7b was generally larger than that of the random copolymer in Figure 7a. These results indicate that the random copolymer can be used as a seed polymer to stabilize the polymerization and as a nanoreactor for emulsion polymerization. However, the diameter according to the TEM micrograph ranged from 100 to 190 nm, smaller than that measured by dynamic light scattering (DLS), as shown in Table 12. The diameter measurement by TEM was conducted in an environment without liquid water and represents the true radius of the particles [61]. On the other hand, the diameter measurement by DLS is conducted in an environment with liquid water and represents the hydrodynamic size of the particles [62,63]. The aggregation state of the particles affects the measured results; thus, the hydrodynamic size of particles or the size of agglomerated particles measured by DLS is often larger than the true radius of particles measured by TEM [63]. Agglomerated particles can be seen in the TEM micrograph (Figure 7); hence, the diameter measured by DLS was larger than that measured by TEM.

We investigated whether the random copolymer can be used as macro-chain transfer agent or macro-emulsifier in the chain extension reaction when BA and HEMA were added in at second polymerization stage. As shown in Table 13, the emulsion of the random copolymer poly (PEGMA-*co*-MAA^a^-*co*-BMA-*co*-MMA-St) was stable and milky white with a weak blue color. When the monomer mixture comprising BA and HEMA was added at the second polymerization stage, the emulsion was not stable, and a lot of coagulation existed after 33 min of reaction time. The pH value of the withdrawn sample ranged between 6.5 and 7, and there were no nonionic MAA monomers that participated in random copolymerization with HEMA, thereby restraining the formation of an interpolymer complex. Crosslinking caused by transesterification among the pendent hydroxyl groups can lead to crosslinked polymers [23,24], and these crosslinked polymers may lead to instability of the emulsion. Thus, the emulsion was not stable, and a large amount of gel existed after 33 min. The instability of the emulsion in the chain extension reaction period indicated that HEMA could not be copolymerized fluently in the chain extension reaction of this emulsion polymerization system.

In conclusion, the living polymerization of the random copolymer chain was proven by the chain extension reaction containing BA and St in the emulsion. Moreover, the copolymer prepared via the chain extension reaction exhibited a higher measured *M*_n_, lower *Ð*, larger particle size, and higher solid content than the random copolymer at the first polymerization stage. The TEM results indicate the increase in particle size and the regular shape of the micelle. However, the chain extension reaction with BA and HEMA was not successful; thus, there exists some limitation to the chain extension reaction of the seed random copolymer with other kinds of monomer.

### 3.6. Infrared Spectra of Polymer

**Figure 8:**
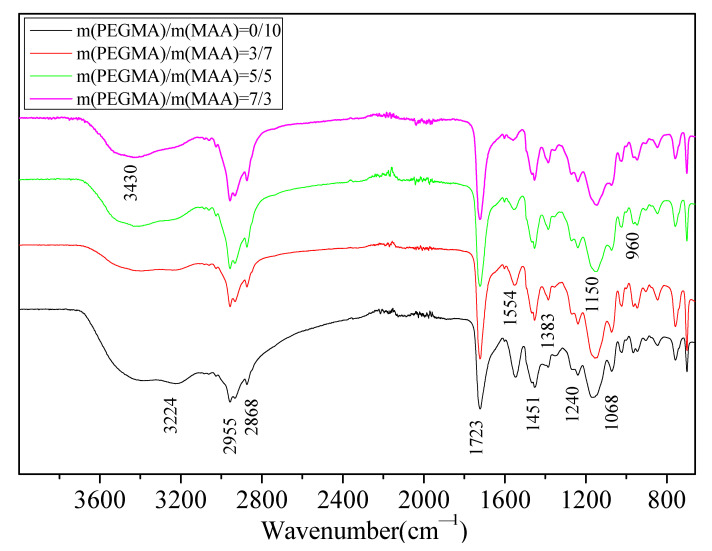
FTIR spectra of copolymer with different mass ratios of PEGMA/MAA. Polymerization conditions: *m*(MAA) + *m*(PEGMA) = 1.664 g; no MMA in the polymerization system; the MAA solution was neutralized by ammonia solution; *m*(BA)/*m*(St) = 1/2; *n*(St)/*n*(HEMA)/*n*(BMA)/*n*(BA)/*n*(ACPA)/*n*(I_2_) = 18.36/18.74/40.36/7.46/1.15/1; the total mass of ingredients without ACPA solution and I_2_ was maintained at 30.08 g in theory.

The FTIR spectra of the copolymer are illustrated in Figure 8. The wide absorption peak at 3430 cm^−1^ was caused by O–H stretching vibration. The wide absorption peak at 3224 cm^−1^ was derived from N–H stretching vibration of NH_4_^+^ located in the ammonium salt of MAA units. The two peaks at 2955 and 2868 cm^−1^ were ascribed to C–H stretching vibrations of –CH_3_ and –CH_2_ groups, respectively. The two peaks at 1451 and 1383 cm^−1^ were caused by C–H bending vibrations of –CH_3_ and –CH_2_ groups, respectively. The strong absorption peak at 1723 cm^−1^ was derived from the stretching vibration of the carbonyl ester C=O. The peak at 1544 cm^−1^ was caused by the asymmetric stretching vibration of carbonyl anion COO^−^. The bands of 1240 and 1068 cm^−1^ were derived from asymmetric stretching vibration and symmetric stretching vibration of the ester group C–O–C, respectively. The strong absorption peak at 1150 cm^−1^ was ascribed to the bending vibration of the ether group C–O–C from PEGMA units and that of C–O–H from HEMA units. With the increase in mass ratio of PEGMA/MAA, the peak intensity at 1544 cm^−1^ was deceased, indicating a decrease in MAA content in the polymer chain. The FTIR spectra indicate the influence of the mass ratio of PEGMA/MAA on the signal changes representing polymeric groups.

## 4. Conclusions

Emulsifier-free styrene–acrylic emulsions prepared via RITP were studied in this paper. When the ammonium salt of MAA was used as the only kind of polymeric surfactant and St was added into the polymerization system containing HEMA monomer component, the emulsion did not flow fluently and was sticky. Thus, it was necessary to explore a method to prepare stable styrene–acrylic emulsions with a high content of HEMA monomer component. In this paper, a stable styrene–acrylic emulsion with low viscosity and an analogous *M*_n_ range to previous work (from 23,000 to 30,000 g·mol^−1^) could be prepared when PEGMA was combined with the ammonium salt of MAA. The acrylate-based polymer emulsion with HEMA units in the polymer chain was not stable when only PEGMA was used as a polymeric emulsifier. St is more hydrophobic than MMA; hence, the polymerization containing St monomer units was conducted in combination with the ammonium salt of MAA and PEGMA. The influence of PEGMA and I_2_ on the emulsion was studied. With the increase in PEGMA amount, the *T*_g_ of the polymer decreased; the largest maximum tensile strength (5.39 MPa) in the polymer with *T*_g_ = 29.9 °C was larger than that of the polyacrylate polymer with *T*_g_ = 43.9 °C (2.98 MPa) in our previous paper. When polymerization was conducted in 1.4 times the reference amount of initiator, *M*_n_ was obviously increased with the decrease in I_2_, and the highest *M*_n_ of the polymer with HEMA units (40,700 g·mol^−1^) was larger than that in our previous paper (32,700 g·mol^−1^) when the mass ratio of BA/BMA was 1/6, while the largest maximum tensile strength of the dried styrene–acrylic emulsion polymer film with the highest *M*_n_ was more than 5.5 MPa. The living polymerization of the random copolymer chain was proven by a kinetics experiment and chain extension reaction; the uniformly shaped particles and the increase in particle diameter according to TEM indicated that the random copolymer can serve as a seed emulsifier in the polymerization. The novelty lies in that the polymerization was conducted with a high solid content (ranging from 40 wt.% to 46 wt.%) and a high content of hydroxyl monomer component, while St took part in the polymerization; a moderate *M*_n_ range (20,000–41,000 g·mol^−1^) was achieved in a short period of time (<4 h), and a stable emulsion with moderate viscosity (ranging from 100 mPa·s to 700 mPa·s) was successfully prepared. The polymerization was conducted in water, and no costly nonpolymeric emulsifier or organic solvent was used. Furthermore, the amount of MAA could be reduced when MAA was combined with PEGMA, and this strengthened the stability of the emulsion when ammonium hydroxide used for neutralizing MAA was volatilized quickly in hot weather. This protocol represents an environmentally friendly system tailored for the direct preparation of an emulsion used for maintenance coating. In summary, the work in this article provides a synthetic method for preparing high-solid-content styrene–acrylic emulsions with HEMA units in the polymer chain, and the synthetic method may have application in preparing coatings or polymer materials with excellent mechanical properties.

## Figures and Tables

**Table 1:** Results of emulsion with St units in polymer chain.

*m*(MMA)/*m*(St)	Conversion (%)	*M*_n,th_^a^ (10^3^ g·mol^−1^)	*M*_n,GPC_ (10^3^ g·mol^−1^)	*Ɖ*	*d*_p_(nm) ^b^/PDI	Viscosity (mPa·s)/Rotor Type/Rotor Rate (rpm)	Reaction Phenomena
0/10	-	-	-	-	-	-	Opalescent, a lot of gel.
4/6	99.5	7.34	18.6	1.89	359/0.139	2350/F/750	Opalescent, high viscosity, no gel.
6/4	>99.5	7.38	24.1	1.70	429/0.040	639/F/750	Opalescent with weak blue color.
8/2	96.8	7.15	27.5	1.56	409/0.042	664/F/750	Opalescent with weak blue color.
10/0	>99.5	7.38	30.2	1.62	357/0.066	525/F/750	Opalescent with weak blue color.

^a^*M*_n,th_ = (mass of monomer) × (monomer conversion)/(2 × *n*_I2_,_initial_) + *M*_AI_, in which *M*_AI_ = 275.02 g·mol^−1^. ^b^ *d*_p_: particle size diameter; PDI: polydispersity index of particle size diameter. Conditions: MAA solution was neutralized by 1.39 g of ammonia solution; *m*(BA)/[*m*(MMA) + m(St)] = 1/2; *m*(MMA) + *m*(St) = 2.054 g; *n*(MAA)/*n*(HEMA)/*n*(BMA)/*n*(BA)/*n*(ACPA)/*n*(I_2_) = 18/18.74/40.36/7.46/1.15/1; the total mass of ingredients without ACPA solution and I_2_ was maintained at 30.08 g in theory.

**Table 2:** Properties of the cured film synthesized by emulsion with St units in polymer chain.

*m*(MMA)/*m*(St)	Pencil Hardness of the Dried Emulsion Film	Pencil Hardness of the Modified Film	Adhesive Property of the Dried Emulsion Film	Adhesive Property of the Modified Film	Toluene Absorption (Wt.%)	Water Absorption (Wt.%)	Water Resistance in Boiled Water
4/6	1 H	2 H	1	0	12.2	5.70	Whitening
6/4	1 H	2 H	1	0	12.0	5.42	Translucent
8/2	1 H	2 H	1	0	11.4	4.47	Whitening
10/0	-	2 H	-	0	16.9	3.82	Translucent

Conditions: MAA solution was neutralized by ammonia solution; *n*(MAA) /*n*(HEMA)/*n*(BMA)/*n*(BA)/*n*(ACPA)/*n*(I_2_) = 18/18.74/40.36/7.46/1.15/1; *m*(BA)/[*m*(MMA) + *m*(St)] = 1/2; *m*(MMA) + *m*(St) = 2.054 g; ammonia solution (1.39 g); the total mass of ingredients without ACPA solution and I_2_ was maintained at 30.08 g in theory.

**Table 3:** Results of emulsion with PEGMA units in polymer chain.

*m*(PEGMA)/*m*(MAA)	Conversion (%)	*M*_n,th_ (10^3^ g·mol^−1^)	*M*_n,GPC_ (10^3^ g·mol^−1^)	*Ɖ*	*d*_p_(nm)/PDI	Viscosity (mPa·s)/Rotor Type/Rotor Rate (rpm)	Reaction Phenomena
0/10	-	-	-	-	-	-	Opalescent, high viscosity, and poor fluidity.
3/7	>99.5	7.14	27.4	1.64	336/0.187	1210/G/750	Opalescent, no gel.
5/5	94.6	6.61	23.3	1.70	271/0.201	151/F/750	Opalescent with weak blue color.
7/3	83.0	5.70	21.0	1.79	220/0.037	38.2/F/750	Opalescent with weak blue color, little white precipitate.

Conditions: *m*(MAA) + *m*(PEGMA) = 1.664 g; no MMA in the emulsion polymerization system; *n*(St)/*n*(HEMA)/*n*(BMA)/*n*(BA)/*n*(ACPA)/*n*(I_2_) = 18.36/18.74/40.36/7.46/1.15/1; *m*(BA)/*m*(St) = 1/2; MAA solution was neutralized by ammonia solution; the total mass of ingredients without ACPA solution and I_2_ was maintained at 30.08 g in theory.

**Table 4:** Properties of the cured film synthesized by emulsion with PEGMA units in polymer chain.

*m*(PEGMA)/*m*(MAA)	Pencil Hardness Rank	Adhesion Property Rank	Water Absorption (Wt.%)
3/7	2 H	1	5.70
5/5	2 H	1	4.69
7/3	2 H	4	2.57

Conditions: *m*(MAA) + *m* (PEGMA) = 1.664 g; no MMA in the polymerization system; *n*(St)/*n*(HEMA)/*n*(BMA)/*n*(BA)/*n*(ACPA)/n(I_2_) = 18.36/18.74/40.36/7.46/1.15/1; *m*(BA)/*m*(St) = 1/2; MAA solution was neutralized by ammonia solution; the total mass of ingredients without ACPA Solution and I_2_ was maintained at 30.08 g in theory.

**Table 5:** Results of emulsion with MMA units in polymer chain.

*m*(MAA)/*m*(PEGMA)	Reaction Time(min)	Conversion (%)	*M*_n,GPC_ (10^3^ g·mol^−1^)	*Ɖ*	*d*_p_(nm)/PDI	Viscosity (mPa·s)/Rotor Type/Rotor Rate (rpm)	Reaction Phenomena
10/0	210	>99.5	30.2	1.62	357/0.066	525/F/750	Milky white with weak blue color.
0/10	320	-	-	-	-	-	Pale yellow color, a lot of flocculation

Conditions: MAA solution was neutralized by ammonia solution; *m*(MAA) + *m*(PEGMA) = 1.664 g; *n*(HEMA)*/n*(BMA)*/n*(BA)*/n*(ACPA)*/n*(I_2_) = 18.74/40.36/7.46/1.15/1; *m*(BA)*/m*(MMA) *=* 1/2; no St in the emulsion; the total mass of ingredients without ACPA solution and I_2_ was maintained at 30.08 g in theory.

**Table 6:** Results of emulsion with MMA and St units in polymer chain.

*m*(MMA)/*m*(St)	Conversion (%)	*M*_n,th_ (10^3^ g·mol^−1^)	*M*_n,GPC_ (10^3^ g·mol^−1^)	*Ɖ*	*d*_p_(nm)/PDI	Viscosity (mPa·s)/Rotor Type/Rotor Rate (rpm)	Solid Content (wt.%)
0/10	>99.5	7.14	27.4	1.64	336/0.187	1210/G/750	45.8
2/8	99.5	7.10	22.7	1.75	340/0.050	454/F/750	41.8
4/6	98.8	7.06	21.3	1.85	333/0.097	46.9/E/750	41.5
6/4	97.6	6.97	22.8	1.73	377/0.062	501/F/750	41.0
8/2	96.2	6.87	27.3	1.57	454/0.249	415/F/750	40.4
10/0	>99.5	7.14	26.5	1.65	430/0.061	1270/G/750	42.6

Conditions: *m*(MMA) + *m*(St) = 2.054 g, *m*(MAA) + *m*(PEGMA) = 1.664 g, and *m*(PEGMA)/*m*(MAA) = 3/7; *n*(MAA)/*n*(PEGMA)/*n*(HEMA)/*n*(BMA)/*n*(BA)/*n*(ACPA)/*n*(I_2_) = 12.60/0.98/18.74/40.36/7.46/1.15/1; ammonia solution (1.10 g); *m*(BA)/[*m*(St) + *m*(MMA)] = 1/2; the total mass of ingredients without ACPA solution and I_2_ was maintained at 30.08 g in theory.

**Table 7:** Properties of the cured film synthesized by emulsion with MMA and St units in polymer chain.

*m*(MMA)/*m*(St)	Pencil Hardness Rank	Adhesive Property Rank	Toluene Absorption (Wt. %)	Water Absorption (Wt. %)	Water Resistance in Boiled Water
0/10	2 H	1	16.0	5.70	Whitening
4/6	1 H	0	13.7	4.05	Whitening
6/4	2 H	0	13.8	2.98	Whitening
8/2	2 H	0	12.8	3.67	Translucent
10/0	3 H	0	12.4	2.28	Translucent

Conditions: *m*(MMA) + *m*(St)= 2.054 g, *m*(MAA) + *m*(PEGMA) = 1.664 g, and *m*(PEGMA)/*m*(MAA) = 3/7; *n*(MAA)/*n*(PEGMA)/*n*(HEMA)/*n*(BMA)/*n*(BA)/*n*(ACPA)/*n*(I_2_) = 12.60/0.98/18.74/40.36/7.46/1.15/1; *m*(BA)/[*m*(St) + *m*(MMA)] = 1/2; ammonia solution (1.10 g); the total mass of ingredients without ACPA solution and I_2_ was maintained at 30.08 g in theory.

**Table 8:** Influence PEGMA on emulsion with St units and MMA units in polymer chain.

*m*(PEGMA)/*m*(MAA)	Conversion (%)	*M*_n,th_ (10^3^ g·mol^−1^)	*M*_n,GPC_ (10^3^ g·mol^−1^)	*Ɖ*	*d*_p_(nm)/PDI	Viscosity (mPa·s)/Rotor Type/Rotor Rate (rpm)	Solid Content (wt.%)
1/7	>99.5	6.98	21.9	1.80	342/0.073	594/F/750	42.7
3/7	98.8	7.20	21.3	1.85	333/0.097	46.9/E/750	41.5
5/7	>99.5	7.29	19.0	1.90	521/0.134	530/F/750	44.4
7/7	>99.5	7.45	21.0	1.84	507/0.176	552/F/750	44.9
9/7	>99.5	7.60	21.3	1.78	442/0.054	620/F/750	45.1
12/7	99.4	7.78	21.9	1.72	439/0.157	767/F/750	46.1

Conditions: *m*(MMA) + *m*(St) = 2.054 g and *m*(MMA)/*m*(St) = 4/6; *m*(MAA) = 1.165 g; *n*(MAA)/*n*(HEMA)/*n*(MMA)/*n*(St)/*n*(BMA)/*n*(BA)/*n*(ACPA)/*n*(I_2_) = 12.60/18.74/7.64/11.02/40.36/7.46/1.15/1; ammonia solution (1.10 g); *m*(BA)/[*m*(St) + *m*(MMA)] = 1/2; the total mass of ingredients without ACPA solution and I_2_ was maintained at 30.08 g in theory.

**Table 9:** Properties of the cured film with different amounts of PEGMA.

*m*(PEGMA)/*m*(MAA)	Pencil Hardness Rank	Adhesive Property Rank	Water Absorption (wt. %)
1/7	2 H	0	3.52
3/7	1 H	0	4.05
5/7	2 H	0	5.03
7/7	2 H	0	7.85
9/7	2 H	0	4.41
12/7	2 H	0	2.89

Conditions: *m*(MMA) + *m*(St) = 2.054 g and *m*(MMA)/*m*(St) = 4/6; *m*(MAA) = 1.165 g; *n*(MAA)/*n*(HEMA)/*n*(MMA)/*n*(St)/*n*(BMA)/*n*(BA)/*n*(ACPA)/*n*(I_2_) = 12.60/18.74/7.64/11.02/40.36/7.46/1.15/1; *m*(BA)/[*m*(St) + *m*(MMA)] = 1/2; ammonia solution (1.10 g); the total mass of ingredients without ACPA solution and I_2_ was maintained at 30.08 g in theory.

**Table 10:** Influence of iodine on emulsion with St units in polymer chain.

*Run*	*m*(I_2_)/*m*(I_2_)_0_	Conversion (%)	*M*_n,th_ (10^3^ g·mol^−1^)	*M*_n,GPC_ (10^3^ g·mol^−1^)	*Ɖ*	*d*_p_(nm)/PDI	Solid Content (wt.%)
1a ^α^	1/1	94.6	6.75	23.3	1.70	271/0.201	39.7
1b ^β^	1/1	>99.5	6.98	19.4	1.97	287/0.052	42.6
2a	4/5	96.4	8.35	24.8	1.71	295/0.194	40.4
3a	3/5	94.6	10.84	24.8	1.63	282/0.107	39.8
3b	3/5	98.5	11.27	32.1	1.45	333/0.096	42.2
4a	2/5	93.5	15.94	26.0	1.58	382/0.181	39.3
5a	1/4	90.4	24.50	27.1	1.50	324/0.093	38.0
5b	1/4	>99.5	27.08	32.9	1.42	373/0.255	43.7

^α^*m*(I_2_)_0_ = 0.273 g; *m*(ACPA) = *m*(ACPA)_0_ = 0.346 g. Conditions: *m*(PEGMA)/*m*(MAA) = 1/1 and *m*(MAA) + *m*(PEGMA) = 1.664 g; no MMA in the polymerization system; *n*(St)/*n*(HEMA)/*n*(BMA)/*n*(BA)/*n*(ACPA)/*n*(I_2_)_0_ = 18.36/18.74/40.36/7.46/1.15/1; *m*(BA)/*m*(St) = 1/2; ammonia solution (0.70 g); the total mass of ingredients without ACPA solution and I_2_ was maintained at 30.08 g in theory. ^β^ *m*(I_2_)_0_ = 0.273 g; *m*(ACPA) = 1.4*m*(ACPA)_0_ = 0.485 g. Conditions: *m*(PEGMA)/*m*(MAA) = 1/1 and *m*(MAA) + *m*(PEGMA) = 1.664 g; no MMA in the polymerization system; *n*(St)/*n*(HEMA)/*n*(BMA)/*n*(BA)/*n*(ACPA)/*n*(I_2_)_0_ = 18.36/18.74/40.36/7.46/1.61/1; *m*(BA)/*m*(St) = 1/2; ammonia solution (0.70 g); the total mass of ingredients without ACPA solution and I_2_ was maintained at 30.08 g in theory.

**Table 11:** Influence of iodine on emulsion with MMA and St units in polymer chain.

*Run*	*m*(I_2_)/*m*(I_2_)_0_	Conversion (%)	*M*_n,th_ (10^3^ g·mol^−1^)	*M*_n,GPC_ (10^3^ g·mol^−1^)	*Ɖ*	*d*_p_(nm)/PDI	Solid Content (wt.%)
1a ^α^	1/1	98.8	7.06	21.3	1.85	333/0.097	41.5
1b ^β^	1/1	>99.5	7.14	21.9	1.81	327/0.088	43.9
2b	3/5	93.3	10.94	25.2	1.66	407/0.074	39.9
3b	1/4	>99.5	27.73	40.7	1.27	403/0.132	43.6

^α^*m*(I_2_)_0_ = 0.273 g; *m*(ACPA) = *m*(ACPA)_0_ = 0.346 g. Conditions: *m*(MMA)/*m*(St) = 4/6 and *m*(MMA) + *m*(St) = 2.054 g; *m*(BA)/[ *m*(MMA) + *m*(St)] = 1/2; *m*(PEGMA)/*m*(MAA) = 3/7; *m*(MAA) + *m*(PEGMA) = 1.664 g; *n*(MAA)/*n*(PEGMA)/*n*(HEMA)/*n*(BMA)/*n*(BA)/*n*(ACPA)/*n*(I_2_)_0_ = 12.60/0.98/18.74/40.36/7.46/1.15/1; ammonia solution (1.10 g); the total mass of ingredients without ACPA solution and I_2_ was maintained at 30.08 g in theory. ^β^ *m*(I_2_)_0_ = 0.273 g; *m*(ACPA) = 1.4*m*(ACPA)_0_ = 0.485 g. Conditions: *m*(MMA)/*m*(St) = 4/6 and *m*(MMA) + *m*(St) = 2.054 g; *m*(BA)/[*m*(MMA) + *m*(St)] = 1/2; *m*(PEGMA)/*m*(MAA) = 3/7; *m*(MAA) + *m*(PEGMA) = 1.664 g; *n*(MAA)/*n*(PEGMA)/*n*(HEMA)/*n*(BMA)/*n*(BA)/*n*(ACPA)/*n*(I_2_)_0_ = 12.60/0.98/18.74/40.36/7.46/1.15/1; ammonia solution (1.10 g); the total mass of ingredients without ACPA solution and I_2_ was kept at 30.08 g in theory.

**Table 12:** Chain extension reaction with BA and St.

Type	Stage Time (min)	Conversion (%)	*M*_n,th_ (10^3^ g·mol^−1^)	*M*_n,GPC_ (10^3^ g·mol^−1^)	*Ɖ*	*d*_p_(nm)/PDI	Solid Content (wt.%)
Seed Polymer Poly (PEGMA-*co*-MAA^a^-*co*-HEMA-*co*-BMA)	130	>99.5	9.05	19.3	1.80	383/0.205	36.0
Block Copolymer Poly (PEGMA-*co*-MAA^a^-*co*-HEMA-*co*-BMA)-*b*-Poly (BA-*co*-St)	90	>99.5	11.44	29.4	1.58	463/0.077	44.6

MAA^a^ was neutralized by ammonia solution. Conditions: *m*(PEGMA)/*m*(MAA) = 1/1 and *m*(MAA) + *m*(PEGMA) = 1.664 g; no MMA in the polymerization system; ammonia solution (0.70 g); *n*(PEGMA)/*n*(MAA)/*n*(St)/*n*(HEMA)/*n*(BMA)/*n*(BA)/*n*(ACPA)/*n*(I_2_) = 2.72/15/18.38/30.62/31.22/67.28/12.44/1.92/1; *m*(BA)/*m*(St) = 1/2; the total mass of ingredients without ACPA solution and I_2_ was maintained at 30.08 g in theory.

**Table 13:** Chain extension reaction with BA and HEMA.

Type	Stage Time (min)	Conversion (%)	Solid Content (wt.%)	Emulsion Appearance
Seed Polymer Poly(PEGMA-*co*-MAA^a^-*co*-BMA-*co*-MMA-St)	168	96.2	38.5	Opalescent with weak blue color.
Block Copolymer Poly(PEGMA-*co*-MAA^a^-*co*-BMA-*co*-MMA-St)-*b*-Poly(PEGMA-*co*-MAA^a^-*co*-BMA-*co*-MMA-St-*co*-BA-*co*-HEMA)	33	-	-	Opalescent, a lot of gel.

MAA^a^ was neutralized by ammonia solution. Conditions: *m*(PEGMA)/*m*(MAA) = 12/7 and *m*(MAA) + *m*(PEGMA) = 3.1611 g; *m*(BA)/[*m*(St) + *m*(MMA)] = 1/2; ammonia solution (1.10 g); *n*(PEGMA)/*n*(MAA)/*n*(St)/*n*(HEMA)/*n*(BMA)/*n*(BA)/*n*(MMA)/*n*(ACPA)/*n*(I_2_) = 6.52/21/18.38/31.22/67.28/12.44/12.74/1.92/1; the total mass of ingredients without ACPA solution and I_2_ was maintained at 30.08 g in theory.
